# Sickness absenteeism among public servants in the Amazon region during the COVID-19 pandemic: a longitudinal study (2019-2022)

**DOI:** 10.47626/1679-4435-2026-1522

**Published:** 2026-02-27

**Authors:** Jessica de Sousa Meneses, Fabrício Augusto Menegon, Lizandra da Silva Menegon

**Affiliations:** 1 Universidade Federal de Santa Catarina, Departamento de Saúde Pública, Florianópolis, SC, Brazil

**Keywords:** pandemic, COVID-19, sickness absence, public sector workers., pandemia, covid-19, licença médica, servidor público.

## Abstract

**Introduction:**

Characterizing sickness absenteeism is essential for understanding the health-disease process within the public sector.

**Objectives:**

To characterize the profile of sickness absence among civil servants during the COVID-19 pandemic period.

**Methods:**

This longitudinal, descriptive study used secondary data from sickness absence records obtained from both the Integrated Civil Servant Health Care Subsystem and the Federal University of Amapá, covering the period from 2019 to 2022. Absences were analyzed according to sex, age group, occupational category, and International Classification of Diseases group.

**Results:**

A total of 675 sickness absences were recorded, corresponding to 12,553 lost workdays, of which 444 were associated with an International Classification of Diseases code. The most frequent diagnostic groups were infectious and parasitic diseases (15.09%), mental and behavioral disorders (12.39%), and diseases of the digestive system (11.49%). Absences were more prevalent among women (68.15%) and among employees aged 30-39 years (36.59%). Administrative staff accounted for the highest proportion of absences (59.09%).

**Conclusions:**

The profile of sickness absenteeism differed from that observed in other public institutions. The findings underscore concerns regarding underreporting and highlight the need to improve institutional communication and to implement targeted strategies aimed at promoting workers’ mental and oral health.

## Resumo

**Introdução:**

Caracterizar o perfil de afastamentos por doença constitui uma estratégia importante para compreender o processo de adoecimento no serviço público.

**Objetivos:**

Analisar o perfil de afastamento do trabalho por motivo de doença na Universidade Federal do Amapá durante os anos da pandemia do coronavírus.

**Métodos:**

Trata-se de um estudo longitudinal e descritivo, com base nos afastamentos por doenças registrados no Subsistema Integrado de Saúde do Servidor Público e na Universidade Federal do Amapá, ocorridos entre 2019 e 2022. Foram analisados os afastamentos por sexo, faixa etária, categoria profissional e código motivador do afastamento, conforme a Classificação Internacional de Doenças.

**Resultados:**

Ocorreram 675 afastamentos, totalizando 12.553 dias perdidos de trabalho e, desses, 444 afastamentos contam com registro da Classificação Internacional de Doenças. As doenças mais frequentes foram as infecciosas e parasitárias (15,09%), os transtornos mentais e comportamentais (12,39%) e as doenças do aparelho digestivo (11,49%). Os afastamentos foram mais presentes entre as mulheres (68,15%) e entre servidores na faixa etária entre 30 e 39 anos (36,59%). A categoria dos técnicos administrativos apresenta a maior ocorrência de afastamentos (59,09%).

**Conclusões:**

O perfil de afastamento apresentou distinção em relação a outros órgãos, e os dados evidenciaram preocupação quanto às subnotificações, às melhorias na comunicação institucional e à necessidade de desenvolver estratégias voltadas para a promoção da saúde mental e bucal dos servidores.

## INTRODUCTION

Characterizing the profile of sickness absenteeism is an important strategy for assessing the health conditions of employees, as it allows the identification of the most frequent diseases and health problems, their causes, and associated factors. This type of approach makes it possible to understand how the work environment, structural conditions, and lifestyle habits influence the health-disease process, in addition to supporting public policies aimed at health promotion and the prevention of work-related diseases.

In the public sector, one of the main data sources for this characterization is the Medical Leave registry within the Integrated Civil Servant Health Care Subsystem (*Subsistema Integrado de Atenção à Saúde do Servidor*, SIASS), in which each case of sickness absence is accompanied by a medical diagnosis coded according to the International Classification of Diseases (ICD). These records allow the examination of the multifactorial nature of work absences, which may be associated with demographic, occupational, organizational, or social factors.

The disease process, however, cannot be dissociated from the prevailing productive model which, by prioritizing results, often imposes precarious and exhausting working conditions. In the public sector, particularly in public education, the increase in working hours, professional devaluation, and the lack of effective occupational health protection policies are key determinants of physical and mental illness. This scenario was further exacerbated by the COVID-19 pandemic, which intensified precariousness, introduced additional challenges related to remote work, and led to an increase in sickness absences, especially due to mental disorders and infectious diseases.

Despite the relevance of this issue, research addressing illness among civil servants remains limited and largely concentrated on specific professional areas, such as nursing and medicine, leaving significant gaps in knowledge regarding other occupational categories.

In this context, the present study aimed to characterize the profile of sickness absenteeism among employees of the Federal University of Amapá (UNIFAP) during the COVID-19 pandemic. Located in one of the most geographically isolated regions of Brazil, the state of Amapá consistently presents social indicators below the national average, particularly in sanitation, public safety, and education. These conditions directly affect the functioning of public institutions, including UNIFAP, which faces persistent structural constraints, limited resources, difficulties in attracting and retaining qualified employees, and reduced investment capacity.

This scenario is further compounded by regional inequalities and the sociocultural complexity of the Amazon region, which demand broader and more integrated institutional responses but often encounter limitations stemming from fragile public management. Such conditions have a direct impact on working environments, contributing to physical and emotional strain and increasing the risk of illness and work absences. Therefore, understanding sickness absenteeism at UNIFAP requires recognizing its insertion within a territory marked by structural vulnerabilities, where regional specificities shape both organizational dynamics and the health of civil servants.

## METHODS

This longitudinal study analyzed sickness absence records from UNIFAP employees between 2019 and 2022. The year 2019 was included as a reference to allow comparisons between the preand post-pandemic periods. Only sick leaves granted for the treatment of the employee’s own health condition were analyzed.

Data were collected from two sources: the SIASS/National Health Foundation database, to which UNIFAP is administratively linked, and UNIFAP’s institutional records. SIASS data were made available in aggregated form and included the number of cases of sickness absence by ICD code, total days of absence, sex, and year. Institutional data from UNIFAP were provided at the individual level in anonymized form and included cases of sickness absence, number of sick days, sex, age, occupational category, and the active workforce size by year.

The integration of these databases revealed structural limitations that directly influenced the choice of statistical techniques. UNIFAP does not provide the ICD code associated with each recorded absence, which restricts diagnostic characterization at the institutional level. Conversely, SIASS does not generate reports stratified by sociodemographic variables beyond sex, providing only the number of cases of sickness absence by sex, year, and ICD code, without distinguishing, for example, the number of distinct employees involved or the occupational category associated with each absence. These discrepancies, combined with recording deficiencies related to the sick leave process, precluded more complex analytical approaches and made a descriptive strategy the most methodologically appropriate option.

Individual-level UNIFAP data were organized into two files: one containing cases of sickness absence and another containing the employee population. Record linkage based on individual identifiers made it possible to track, over time, the frequency and recurrence of absences among employees.

Statistical analyses were performed using Stata®. Simple frequencies, proportions, and measures of central tendency and dispersion were calculated. Temporal trends were examined on a year-by-year basis. Annual prevalence was estimated by dividing the number of employees with at least one recorded sickness absence by the total number of active employees in each year, multiplied by 100, with stratification by sex and occupational category.

In analyses by ICD group, measures of central tendency (mean, median, and SD) were applied only to the variable “total days of absence.” This methodological decision reflects the structure of the database, which is organized by combinations of year, sex, and ICD chapter, rendering the estimation of central tendency measures for the variable “number of cases of sickness absence” inappropriate and potentially misleading. Accordingly, analyses were conducted to preserve statistical consistency and the epidemiological robustness of the findings.

The study was conducted in accordance with National Health Council Resolutions No. 510/16 and No. 580/18 on ethics in Social Sciences and Humanities and was approved by the UNIFAP Research Ethics Committee, under CAAE No. 81540124.4.0000.000 and approval opinion No. 7,105,243.

## RESULTS

Between 2019 and 2022, UNIFAP recorded 675 cases of sickness absence, according to Table 1, totaling 12,553 lost workdays. Over this period, the volume of absences did not follow a linear trajectory. In 2019, 279 cases were recorded, representing the highest number in the series. In the subsequent two years, already under the direct impact of the COVID-19 pandemic, there was a marked reduction, with 101 absences in 2020 and 94 in 2021. In 2022, the number increased again, reaching 201 absences - more than double the figure observed in the previous year -, suggesting a rebound in demand for sick leave following the period of greatest restriction.

**Table 1:** Description of work absences according to sex, age group, and occupational category at the Federal University of Amapá (2019-2022)

Variables	2019		2020		2021		2022		Total	
	n	%	n	%	N	%	n	%	n	%
Sex										
Female	191	68.46	60	59.41	57	60.64	152	75.62	460	68.15
Male	88	31.54	41	40.59	37	39.36	49	24.38	215	31.85
Category										
Academic staff	72	25.81	30	29.70	59	62.77	73	36.32	234	34.67
Administrative staff	207	74.19	71	70.30	35	37.23	128	63.68	441	65.33
Age group (years)										
20 to 29	35	12.54	11	10.89	5	5.32	5	2.49	56	8.30
30 to 39	119	42.66	34	33.66	34	36.17	60	29.8	247	36.59
40 to 49	61	21.86	26	25.74	33	35.11	96	47.76	216	32.00
50 to 59	39	13.98	23	22.77	14	14.89	26	12.94	102	15.11
60 >	25	8.96	7	6.93	8	8.51	14	6.97	54	8.00
TOTAL	279	100	101	100	94	100	201	100	675	100

Women accounted for most cases (68.15%). Among men (31.85%), a notable pattern emerged in 2022, when they represented more than three-quarters of recorded absences. Regarding occupational category, administrative staff accounted for 65.33% of records, whereas academic staff represented 34.67%. Only in 2021 did academic staff predominate, accounting for 62.77% of absences. In terms of age distribution, sickness absence was most prevalent among employees aged 30-39 years (36.59%) and 40-49 years (32.00%), followed by those aged 50-59 years (15.11%), 20-29 years (8.30%), and 60 years or older (8.00%), indicating a concentration of illness primarily among middle-aged workers.

**Figure 1:**
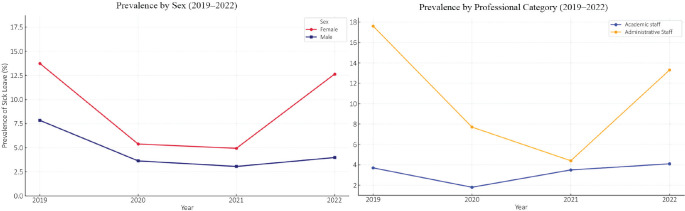
Prevalence of sickness absences by sex and occupational category at the Federal University of Amapá (2019-2022).

The prevalence of sickness absence was higher among women in all years analyzed, with notable differences in 2019 (13.7% vs. 6.7%) and 2022 (12.6% vs. 4.0%). Administrative staff consistently presented higher prevalence rates than academic staff (2019: 17.6% vs. 3.7%; 2022: 13.3% vs. 4.1%). The trend in the prevalence of sick leave according to sex and occupational category is illustrated in Figure 1. Age-stratified analysis revealed an overall decline in 2020, followed by recovery from 2021 onward, particularly among individuals aged 40-49 and 50-59 years. By the end of the study period, sickness absence rates had decreased among those aged 20-29 (-75.5%), 30-39 (-24.3%), and 40-49 years (-28.7%), but increased among employees aged 50-59 years (+17.0%) and, most markedly, 60-75 years (+193.5%), based on the data in Table 1.

Women exhibited a higher mean number of cases of sickness absence (3.92) compared with men (3.17), as well as a higher mean number of lost workdays (65.36 vs. 56.40 days), although the median number of sick days per employee was similar between sexes (30 days). Greater variability in sickness absence duration was also observed among women, as reflected by a higher SD. Regarding occupational category, administrative staff showed a higher mean number of cases of sickness absence per employee (3.77), whereas academic staff accumulated a longer mean duration of sickness absence (83.79 days), with a median of 43 days, exceeding that observed among administrative staff (26 days). These findings indicate that, although administrative staff tend to be absent more frequently, academic staff experience longer periods of absence, reflecting distinct absenteeism patterns.

Analyses by ICD group showed that groups S (1,654 days; median = 55), F (1,279 days; median = 22), and C (1,050 days; median = 52.5) accounted for the highest total number of lost workdays, indicating more prolonged absences. In terms of frequency, the groups with the highest number of records were A-U (67), F (54), and K (51). However, Groups A-U (median = 9.5) and K (median = 7) were associated with lower median numbers of sick days, indicating predominantly short absences, whereas Group F stood out for simultaneously ranking among the groups with the highest number of cases of sickness absence and those with the highest number of total days of absence (Table 2).

**Table 2:** Measures of central tendency for the duration of absences by ICD-10 group at the Federal University of Amapá (2019-2022)

ICD group	No.	Mean (days)	Median (days)	SD (days)	Total (days)
C	26	65.63	52.5	54.09	1.050
S	50	55.13	55	50.43	1.654
I	18	39.92	30	35.24	479
A-U	67	36.96	9.5	48.03	887
F	54	32.79	22	30.89	1.279
M	31	27.3	10	35.87	737
O	9	24.88	23.5	16.75	199
E	9	19.57	18	16.22	137
G	7	17.71	5	22.21	124
L	3	16.67	16	13.01	50
H	19	16	8	20.4	240
J	43	15.97	5	38.68	607
K	51	15.61	7	18.28	687
R	17	13.05	5	20.19	222
N	17	11.76	10	11.45	200
Z	19	10.21	5	14.2	194

ICD = International Classification of Diseases.

Measures of central tendency were not applied to the variable “number of cases of sickness absence” by diagnostic group, as this information is aggregated by combinations of year, sex, and ICD chapter and does not represent individual units of analysis. Consequently, the use of means or SD would yield methodologically inappropriate estimates.

## DISCUSSION

At UNIFAP, women accounted for most cases of sickness absence (68.15%), as shown in Table 1, a finding that can be explained by a combination of biological, social, and cultural factors. Gender socialization directly influences patterns of illness, as the accumulation of daily activities tends to place a greater physical and mental burden on women, increasing their vulnerability to health problems. In addition, women generally seek health care more frequently than men, which reduces presenteeism and, consequently, increases the formal reporting of sickness absence. The unequal division of domestic work and childcare responsibilities also plays a significant role in this process [1].

This pattern is reflected in national data. In 2019, women spent, on average, 10.4 more hours per week than men on domestic and caregiving activities, a disparity that remained substantial in 2022, at 9.6 hours [2]. Although the National Household Sample Survey (*Pesquisa Nacional por Amostra de Domicílios*, PNAD) was not conducted in 2020 and 2021, it is reasonable to assume that social distancing measures during this period intensified these demands, contributing to the worsening of physical, mental, and emotional exhaustion [3].

This interpretation is further supported by studies indicating that the COVID-19 pandemic deepened social, economic, and gender inequalities, significantly affecting women’s daily lives - particularly those who simultaneously occupy the roles of mothers, teachers, and researchers. These women reported increased fatigue, work overload, and greater difficulty in reconciling motherhood with academic work during the pandemic period [4].

Patterns of illness are also shaped by occupational category. Although academic staff constitute the majority of UNIFAP’s workforce, administrative staff accounted for a higher number of cases of sickness absence during the study period. This difference may be explained by distinct work arrangements: whereas academic staff, due to the relative flexibility of teaching, research, and extension activities, often continue to perform part of their duties even while sick, administrative staff are subject to more rigid routines of monitoring and record-keeping, which favors the formalization of absences [1,5,6]. Moreover, academic staff absences entail complex consequences, such as the need to reschedule classes and manage accumulated academic tasks, which may discourage the reporting of non-disabling health conditions [7,8].

This differentiation became even more pronounced in 2020, when the pandemic led to the suspension of on-site activities [9-11]. While administrative staff rapidly transitioned to remote work, supported by the implementation of supervised work plans and activity reports, academic staff remained engaged in discussions regarding pedagogical reorganization, with classes resuming only in November following the adoption of remote teaching modalities [12-14]. Nevertheless, even during this period, academic work was not fully interrupted, as research, extension activities, academic supervision, and scientific production continued to be carried out.

The local context was further aggravated by the energy blackout that occurred in November 2020, which affected approximately 90% of the population of Amapá and once again disrupted the supplementary academic calendar. The combination of class suspension, the adoption of remote work, and the energy crisis likely contributed to the underreporting of absences, particularly among academic staff. Thus, the observed reduction in recorded cases of sickness absence may not necessarily reflect a lower incidence of illness, but rather difficulties in recognizing and formally recording health conditions, reinforcing previous findings regarding the limitations of administrative statistics during periods of crisis [15-17].

**Figure 2:**
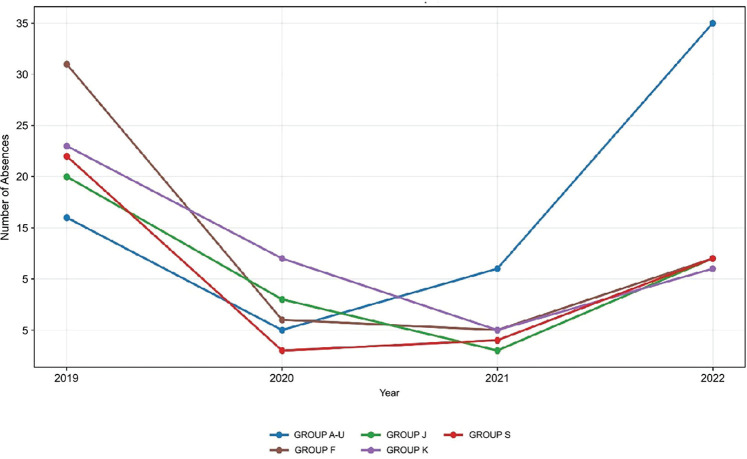
Temporal trends in the five most frequent ICD-10 groups associated with sickness absences at the Federal University of Amapá (2019-2022).

Although UNIFAP recorded 675 cases of sickness absence during the study period, SIASS had only 444 cases associated with an ICD code, revealing a substantial discrepancy between the two data sources. In the analysis by ICD group, the most frequent were A-U (15.09%), largely influenced by COVID-19; F (12.39%), mental and behavioral disorders; K (11.49%), diseases of the digestive system; J (9.68%), respiratory diseases; and S (9.23%), injuries and trauma. The remaining ICD chapters collectively accounted for 42.12% of recorded absences. The temporal evolution of the five most frequent ICD-10 groups can be observed in Figure 2.

ICD A-U: infectious and parasitic diseases (A) and codes for special purposes (U); ICD F: mental and behavioral disorders; ICD K: diseases of the digestive system; ICD J: diseases of the respiratory system; ICD S: injuries and trauma; Other: ICD C, E, G, H, I, L, M, N.

National studies on sickness absenteeism in federal higher education institutions (FHEI) have documented epidemiological patterns that, while sharing certain thematic similarities, also differ substantially from the profile observed at UNIFAP, particularly due to differences in the temporal scope of analysis. Most of the studies reviewed - conducted at the Federal University of Mato Grosso do Sul (2014-2018), Federal University of Maranhão (2011-2013), Federal University of Rio Grande do Norte (2016), Federal University of the São Francisco Valley (2010-2015), Federal University of Pelotas/Federal University of Rio Grande (2015-2019), and Federal University of the Jequitinhonha and Mucuri Valleys (2015-2019) - cover periods prior to the COVID-19 pandemic, during which ICD chapters A-U and J accounted for a smaller proportion of cases of sickness absence due to the lower circulation of infectious and respiratory diseases [18].

In contrast, only a limited number of studies included pandemic years, such as those conducted at the Federal Institute of Roraima (IFRR) (2018-2022), the Federal University of ABC (2020-2021), and the Federal University of Espírito Santo (UFES) (2012-2022). These institutions reported marked changes in sickness patterns, including an increase in diagnoses classified under ICD group A-U. Another important aspect observed in these FHEIs, as well as at UNIFAP, was a reduction in the number of recorded cases during the initial years of the pandemic, followed by a subsequent increase, associated with changes in work organization, exposure to the virus, and demand for healthcare services [18]. At UNIFAP, this fluctuation was evident in the decline observed between 2019 and 2021 (279, 101, and 94 cases, respectively) and the increase in 2022 (201 cases). Similar trends were identified at IFRR and UFES, albeit with differing magnitudes [18].

Furthermore, UNIFAP’s profile regarding the underlying causes of sickness absence also differed from that reported in other FHEIs, both in terms of the predominant health conditions and their distribution across ICD groups. While most institutions reported mental and behavioral disorders (ICD F) and musculoskeletal diseases (ICD M) as the leading causes of sickness absence [18], UNIFAP exhibited a distinct pattern in which ICD F and ICD K (diseases of the digestive system) ranked among the most prevalent groups - a phenomenon not observed in the other FHEIs. It is nevertheless acknowledged that during pandemic years, conditions classified under ICD groups A-U (COVID-19 and other viral infections) assumed a predominant position at both UNIFAP and other FHEIs, reflecting the broader health impact of the period.

This uncommon finding suggests the need for more in-depth investigations to better understand the potential determinants underlying this pattern, including possible links to the structural, sanitary, and socioeconomic vulnerabilities of the state of Amapá. Such specificity may reflect disparities in access to health services, regional inequalities, limitations in dental care provision, or the interplay of multiple factors that warrant further exploration in future studies.

A marked underreporting of COVID-19 cases was identified when comparing SIASS records (47 cases) with data from UNIFAP’s Quality of Life Division, which documented 75 monitored employees (including active and retired staff) during 2020 and 2021. Among active employees, four died from COVID-19 - three academic staff members and one administrative staff member; among the academics, two were also healthcare professionals [19]. Because these cases did not result in formally registered sick leaves, they are absent from the SIASS database, highlighting that a portion of employees became sick - and even died - without these cases being captured by the institutional health surveillance systems.

This discrepancy reveals significant structural limitations in the processes of employee health recording and surveillance, generating gaps that compromise the completeness, sensitivity, and analytical capacity of official databases. Reliance on a single data source, in this case SIASS, may underestimate and distort the true magnitude of illness within institutions. Such weaknesses become even more critical during a pandemic, a period in which rapid case identification, consistent record-keeping, and effective intersectoral data integration are essential to guide protective measures and worker support.

In 2021, the implementation of the *SouGov* application allowed the electronic submission of sick leave medical certificates and the scheduling of medical evaluations, representing an important advance in this process. Nevertheless, the findings presented here indicate that systemic and organizational weaknesses persist and must be addressed to improve institutional health surveillance.

Prior to *SouGov*, the registration process was entirely in-person: employees were required to attend SIASS facilities to schedule and subsequently undergo medical evaluation. This requirement frequently hindered the formal recording of sickness absence among staff from geographically distant campuses, such as the Oiapoque campus, reinforcing the structural nature of the underreporting identified [18].

Additionally, the implementation of medical evaluation based on documentary review, allowing sick leave to be granted without in-person evaluation, also contributed to increasing the formalization of absences, particularly by reducing logistical barriers [18]. However, although technological advances represent an important step toward improving record quality and simplifying administrative workflows, the results of this study demonstrate the continued need for greater integration between data systems, improved institutional communication, and mechanisms to ensure the complete, timely, and standardized capture of health events. Such measures are essential to strengthen worker health surveillance and enhance the reliability of data used for epidemiological analyses.

Analysis of lost workdays due to illness shows that injuries and trauma (ICD S), neoplasms (ICD C), and mental and behavioral disorders (ICD F) accounted for the longest durations of absence, a finding consistent with the clinical complexity of these conditions, which often require prolonged treatment, intensive follow-up, and extended recovery periods [1]. In the specific case of ICD F, in addition to longer periods of sickness absence, a high number of records was also observed - an outcome that aligns with national literature and with the substantial increase in mental health problems during the study period [1,15-18]. The prolonged duration of work absences due to mental disorders may be associated with their multifactorial nature, high risk of recurrence, and the need for continuous monitoring, aspects that could not be explored in greater depth due to limitations of the SIASS database, which does not allow the identification of individual recurrence.

The limitations of this study are concentrated in three main aspects: (i) the limited availability of variables and the fragility of record systems (for example, SIASS relies on outdated infrastructure and offers limited reporting capabilities); (ii) changes in work organization, particularly among academic staff, who adopted greater autonomy and hybrid work arrangements, making it more difficult to identify short-term absences; and (iii) the severity of the pandemic itself, which - due to the prioritization of COVID-19 and the implementation of isolation policies - may have led employees to avoid seeking health care and reporting other causes of illness. These factors indicate that the observed reduction in sickness absence during the study period should be interpreted with caution, as it may reflect methodological and contextual constraints rather than a genuine decline in morbidity.

## CONCLUSIONS

This study examined sickness absence at UNIFAP between 2019 and 2022 and demonstrated that illness in the public sector is a multifactorial phenomenon, shaped by gender inequalities as well as by work organization and geographic location. The weaknesses identified, such as the absence of key variables in the SIASS database, restrictions on data extraction, and underreporting, particularly during periods of remote work, highlight the challenges inherent in using secondary data sources and draw attention to their ethical, technical, and political implications within the fields of public and occupational health.

The findings point to practical avenues for strengthening institutional employee health policies. These include the need to improve communication flows, record-keeping, and integration across different information systems; to invest in permanent mental health initiatives, including preventive actions and psychosocial support; to expand attention to digestive health, particularly oral health, in light of the high frequency of ICD K conditions; and to review management practices that tend to normalize presenteeism and continued work activity despite signs of illness. The development of continuous surveillance tools, such as epidemiological bulletins, observatories, and technical reports, also emerges as essential to support decision-making and guide more timely and effective interventions.

By presenting novel data from the Amazon region and discussing how territorial inequalities, weaknesses in information systems, and the effects of the pandemic shape patterns of illness, this study engages with broader debates on occupational health in the federal public service. It also contributes methodologically by explicitly addressing the limits and potentialities of using secondary databases, a topic that remains insufficiently explored in a critical manner in the existing literature.

In summary, the results reinforce the importance of health policies that combine care-oriented actions, improved information quality, and a critical reassessment of work management models. By bringing together empirical evidence, institutional analysis, and actionable recommendations, this study seeks not only to inform improvements at UNIFAP, but also to broaden the dialogue on more equitable and effective practices for health promotion within the public sector.
